# Reusable UV-crosslinked MIL-101(Fe)@CQD/polymer nanocomposite films for fluorescence detection of As(iii) in water

**DOI:** 10.1039/d6ra08414a

**Published:** 2026-10-23

**Authors:** Yunus Emre Toprak, Soner Çubuk, Memet Vezir Kahraman

**Affiliations:** a Department of Chemistry, Faculty of Science, Marmara University 34722 Istanbul Türkiye mvezir@marmara.edu.tr

## Abstract

The sensitive determination of trace As(iii) in environmental waters remains challenging, despite the availability of sophisticated instrumental techniques. Herein, a free-standing solid-state fluorescence sensing film was fabricated by immobilizing a MIL-101(Fe)@CQDs composite within a UV-curable polymer matrix. Structural, morphological, optical, and surface analyses using FTIR, XRD, UV-Vis spectroscopy, BET, SEM/STEM, DLS, and XPS supported the formation of the composite and its incorporation into the polymer network. The synthesized CQDs exhibited a photoluminescence quantum yield of 19.2 ± 3.2% (*n* = 3). Quantitative determination was based on the fluorescence-intensity difference (Δ*I* = *I*_0_ − *I*). The calibration response was linear over 4.44 × 10^−9^–6.67 × 10^−8^ mol L^−1^ (*R*^2^ = 0.9919), with an LOD of 1.49 × 10^−9^ mol L^−1^ and a calculated LOQ of 4.97 × 10^−9^ mol L^−1^. The method showed high tolerance toward the tested potentially interfering species, including As(v), Sb(iii), Sb(v), carbonate, bicarbonate, phosphate, and common metal ions. Practical applicability was evaluated using environmental water samples and certified reference materials for arsenic, yielding recoveries of 101.3–104.6% with relative standard deviations below 5%. Results for environmental water samples were consistent with direct HG-AAS performed without a separate As(v)-to-As(iii) pre-reduction step; this comparison was used as supporting evidence of analytical consistency rather than as proof of exclusive As(iii) detection. The same sensing film was reused over 150 measurement/regeneration cycles, with a 1.4% difference between the first and final responses. The combined MIL-101(Fe), CQD, and UV-curable polymer platform therefore provides an easy-to-handle and reusable solid-state format for trace As(iii) determination in aqueous samples.

## Abstract 2

The sensitive determination of trace As(iii) in environmental waters remains challenging, despite the availability of sophisticated instrumental techniques.

## Graphical abstract

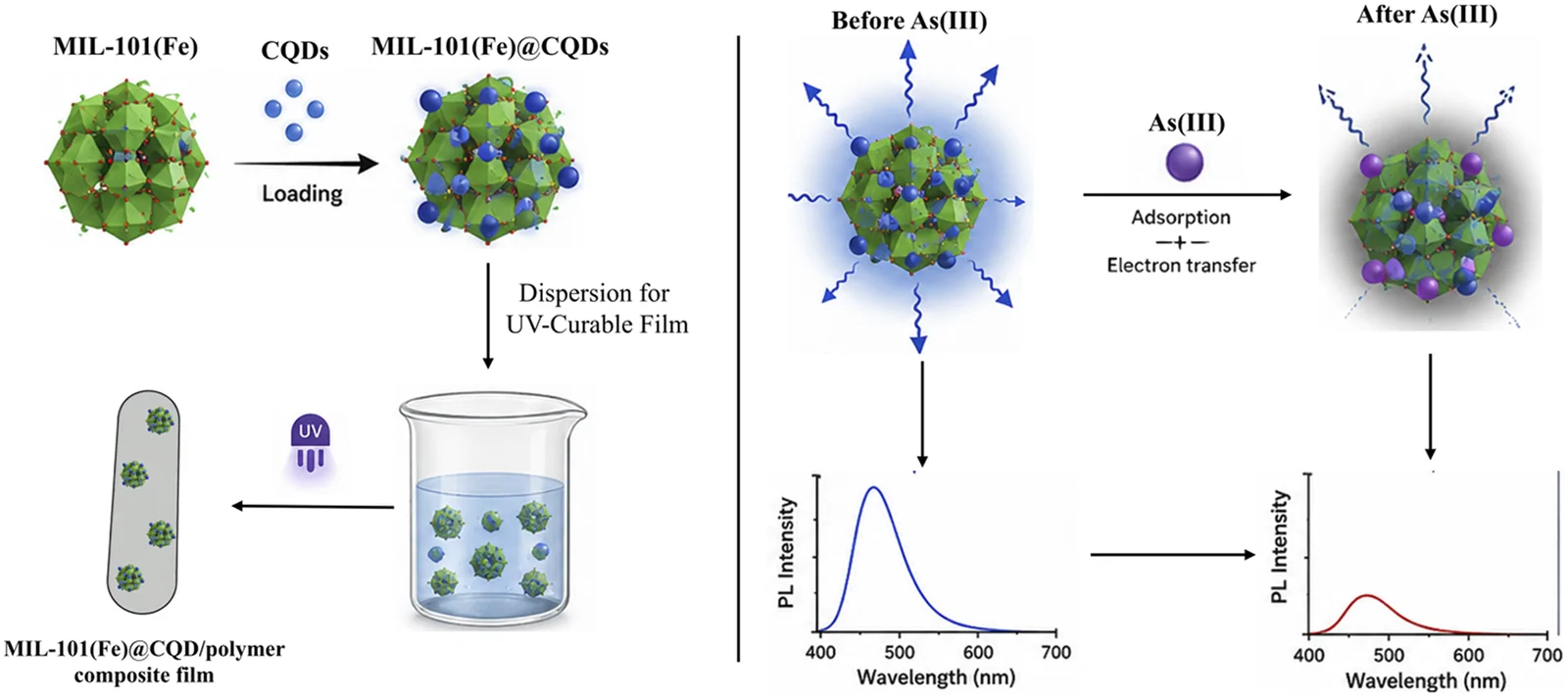

## 1. Introduction

Arsenic contamination of water resources has become one of the most pressing environmental and public health concerns worldwide because of its toxicity, persistence, and carcinogenicity.^1,2^ Chronic exposure to arsenic through contaminated drinking water has been linked to a broad range of adverse health effects, including cardiovascular disorders, neurological dysfunction, and characteristic skin lesions.^1–3^ Increased risks of several cancers have also been reported with chronic arsenic exposure.^4,5^ In response to these health risks, the World Health Organization (WHO) has established a provisional guideline value of 10 µg L^−1^ for arsenic in drinking water.^6^

Conventional analytical techniques, including inductively coupled plasma mass spectrometry (ICP-MS) and atomic absorption spectroscopy (AAS), provide high analytical sensitivity and accuracy for arsenic determination.^7,8^ Nevertheless, these techniques require expensive instrumentation, labor-intensive sample preparation, and trained personnel, limiting rapid on-site monitoring.^9^ In contrast, fluorescence-based sensing strategies offer operational simplicity, rapid response, low detection limits, and compatibility with portable analytical devices.^10^ Fluorescent nanomaterial platforms have also been extended to diverse aqueous analytes and to portable or point-of-care formats, including the detection of organic contaminants.^11^

Among the numerous fluorescent nanomaterials investigated in recent years, carbon quantum dots (CQDs) have attracted significant interest for their photoluminescence.^12^ CQDs have also been successfully incorporated into reusable polymeric sensing architectures.^13^ Their high aqueous dispersibility^14^ and low cytotoxicity,^15^ together with their broad use in bioanalytical detection and imaging,^16^ have contributed to their appeal as optical nanomaterials. Their surfaces are rich in oxygen- and nitrogen-containing functional groups, including hydroxyl, carboxyl, carbonyl, and amino moieties, which enhance water compatibility and provide active sites for interaction with target analytes.^17^ These physicochemical characteristics have enabled CQDs to find applications in photocatalysis, bioimaging, chemical sensing, and environmental remediation.^18^

In this study, CQDs were synthesized hydrothermally using citric acid and ethylenediamine as chemically defined carbon and nitrogen precursors, respectively. This route was selected to provide a defined precursor composition and to introduce nitrogen-containing surface functionalities relevant to the optical response and interfacial interactions of the CQDs. Although green and biogenic synthesis approaches offer potential sustainability benefits, the present study does not aim to establish the superiority of the selected chemical route through a direct comparison. Instead, the main novelty lies in integrating the synthesized CQDs with MIL-101(Fe) and immobilizing the resulting composite in a UV-crosslinked polymer film to develop a reusable solid-state fluorescence sensor for As(iii) determination in water.

Metal–organic frameworks (MOFs) are an important class of functional materials owing to their high specific surface areas, well-defined pore architectures, tunable compositions, and accessible coordination sites.^19,20^ Among these materials, MIL-101(Fe) has attracted particular attention for its hydrothermal stability, iron-based coordination environment, and affinity for arsenic-containing species.^21^ Coordinatively unsaturated Fe(iii) sites can participate in surface complexation with arsenic species, thereby promoting adsorption and local enrichment near the sensing interface.^22^ These characteristics make MIL-101(Fe) a useful host material for chemical sensing and related analytical platforms.^23^

Integrating fluorescent carbon quantum dots (CQDs) with porous MOFs has emerged as a strategy to combine analyte enrichment with optical signal transduction.^24^ In these hybrid materials, the MOF can limit CQD aggregation and increase contact between the analyte and fluorescent domains, whereas CQDs convert changes at the sensing interface into measurable fluorescence responses.^25^ CQDs are also well established as sensing and sorbent materials in environmental analysis,^26^ and recent studies have highlighted CQD@MOF architectures for luminescence regulation and sensing applications.^27^ Such composites may therefore provide complementary analyte-enrichment and optical-transduction functions, depending on composite architecture and measurement conditions.

Fluorescent MOF-based sensing systems have increasingly been developed in device-oriented formats.^28^ MOF-based analytical devices more broadly demonstrate the feasibility of incorporating porous materials into structured analytical platforms,^29^ while polymeric sensing architectures are widely used to improve sensor handling and integration.^30^ Film-based fluorescent materials are especially attractive because they reduce particle handling and enable compact optical readout.^31^ MOF/polymer composites provide an additional route to immobilizing functional materials.^32^ Nevertheless, UV-curable polymer films containing MIL-101(Fe)@CQDs composites for As(iii) determination remain comparatively underexplored, and the relationships among composite structure, optical response, and analytical performance require further study.

In this study, MIL-101(Fe)@CQDs was prepared by solution adsorption and immobilized within a UV-curable polymer matrix to produce a free-standing fluorescence sensing film for As(iii) determination. MIL-101(Fe) provides adsorption sites near the fluorescent CQDs, which serve as optical transducers. Structural, morphological, optical, and surface analyses were used to evaluate composite formation and film incorporation. The analytical response was examined through pH optimization, calibration, response-time measurements, selectivity and interference studies, environmental water analysis, comparison with direct HG-AAS, and validation using certified reference materials for arsenic.

## 2. Experimental

### 2.1. Reagents and materials

Iron(iii) chloride hexahydrate (FeCl_3_·6H_2_O), terephthalic acid (H_2_BDC), *N*,*N*-dimethylformamide (DMF), ethanol, citric acid, ethylenediamine, sodium acetate, acetic acid, and other analytical-grade reagents used in this study were obtained from Sigma–Aldrich (Germany). Ultrapure water with a resistivity of 18.2 MΩ cm was supplied by a Milli-Q purification system (Millipore, USA). Stock solutions of As(v), Sb(iii), Sb(v), carbonate, bicarbonate, and phosphate were prepared from the corresponding analytical-grade sodium salts. Certified reference materials included the NASS-5 seawater reference material (National Research Council Canada), Seronorm Trace Elements in Serum, Level 2 (Sero; supplied by LGC Standards, product code SERO203105), and the SPS-WW1 and SPS-WW2 wastewater reference materials (Spectrapure Standards AS, Oslo, Norway).

### 2.2. Analytical instrumentation

The structural and optical properties of the synthesized materials were characterized by Fourier-transform infrared (FTIR) spectroscopy on a PerkinElmer Spectrum 100 instrument, UV-Vis absorption spectroscopy on a Shimadzu UV-1800 spectrophotometer, and photoluminescence spectroscopy on a Varian Cary Eclipse spectrofluorometer.

The crystal structure was evaluated by X-ray diffraction (XRD) on a Philips diffractometer using Cu Kα radiation (*λ* = 1.5406 Å). Morphology and particle architecture were examined using a Thermo Scientific Quattro S scanning electron microscope equipped with a STEM detector. Dynamic light scattering (DLS) and zeta-potential measurements were performed on Malvern Zetasizer and Brookhaven 90Plus instruments.

Textural properties were evaluated by nitrogen adsorption using the Brunauer–Emmett–Teller (BET) method. Surface elemental composition and chemical states were examined by X-ray photoelectron spectroscopy (XPS) on a Thermo Fisher Scientific K-Alpha spectrometer. XPS binding energies were charge-corrected by assigning the C 1s C–C/C=C component to 284.8 eV. Fluorescence spectra of the polymer films were recorded on the Varian Cary Eclipse spectrofluorometer. Comparative measurements were performed by HG-AAS on an Analytik Jena ZEEnit 700 P instrument equipped with a hydride-generation system; arsenic absorbance was monitored at 193.7 nm with a 0.8 nm spectral slit width.

### 2.3. Synthesis of CQDs

Carbon quantum dots (CQDs) were synthesized *via* a hydrothermal method using citric acid and ethylenediamine as the carbon and nitrogen precursors, respectively, based on previously reported procedures with modifications.^33^ Briefly, 1.05 g of citric acid and 335 µL of ethylenediamine were dissolved in 20 mL of deionized water under continuous magnetic stirring until a transparent precursor solution was obtained. The resulting solution was transferred into a Teflon-lined stainless-steel autoclave and hydrothermally treated at 200 °C for 8 h to promote the formation of fluorescent CQDs. After naturally cooling to room temperature, the obtained CQD dispersion was subjected to the subsequent purification procedure.

After the reaction, the autoclave was allowed to cool naturally to room temperature. The resulting colloidal suspension was purified by centrifugation at 12 000 rpm for 15 min to remove large carbonaceous aggregates. The supernatant was then filtered through a 0.22 µm membrane, followed by dialysis against deionized water using a 1 kDa molecular-weight-cutoff membrane for 24 h, with the external water refreshed periodically to ensure efficient removal of residual precursors and low-molecular-weight by-products. Finally, the purified CQD dispersion was concentrated by vacuum drying at 60 °C, yielding solid CQDs that were stored in the dark until further use.

### 2.4. Synthesis of MIL-101(Fe)

MIL-101(Fe) was synthesized *via* a solvothermal self-assembly method based on previously reported procedures with modifications.^34^ Briefly, FeCl_3_·6H_2_O (2.45 mmol) and terephthalic acid (H_2_BDC, 1.24 mmol) were dissolved in 15 mL of *N*,*N*-dimethylformamide (DMF) under continuous stirring until a homogeneous precursor solution was obtained. The resulting solution was transferred into a Teflon-lined stainless-steel autoclave and heated at 170 °C for 24 h to promote the solvothermal crystallization of the MIL-101(Fe) framework. After naturally cooling to room temperature, the resulting solid was collected and subjected to the subsequent washing and activation procedures.

After the solvothermal treatment, the reactor was allowed to cool to ambient temperature. The solid product was collected by centrifugation and purified by repeated washing with DMF to remove residual linker molecules, followed by several washes with absolute ethanol to remove the remaining solvent. The purified MIL-101(Fe) crystals were finally dried under reduced pressure at 60 °C for 24 h and stored in a desiccator prior to subsequent composite preparation and characterization.

### 2.5. Preparation of MIL-101(Fe)@CQDs

The MIL-101(Fe)@CQDs composite was prepared by a solution-based adsorption method, based on previously reported approaches for decorating MIL-101(Fe) with carbon quantum dots, with modifications.^35^ Briefly, activated MIL-101(Fe) (100 mg) was dispersed in 50 mL of an aqueous CQD suspension containing 50 mg of CQDs. The resulting dispersion was continuously stirred at room temperature for 24 h to facilitate the adsorption and interfacial association of CQDs onto the MIL-101(Fe) surface. The resulting MIL-101(Fe)@CQDs composite was subsequently separated and subjected to the appropriate washing and drying procedures.

After adsorption, the composite was collected by centrifugation and repeatedly washed with absolute ethanol to remove loosely associated CQDs. The resulting MIL-101(Fe)@CQDs composite was then dried under reduced pressure at 60 °C overnight and stored in a desiccator until film fabrication.

### 2.6. Determination of photoluminescence quantum yield

The photoluminescence quantum yield (PLQY) of the synthesized CQDs was determined by a relative method using quinine sulfate as the reference fluorophore. Quinine sulfate in 0.1 M H_2_SO_4_ was used as the reference because of its established quantum yield (*Φ* = 0.54) at an excitation wavelength of 360 nm. Fluorescence emission spectra of the CQD dispersion and the reference solution were recorded under identical instrumental conditions, and the integrated area of each spectrum was calculated over the same wavelength interval encompassing the complete emission band centered near 450 nm. To minimize inner-filter effects, the absorbance of both solutions at the excitation wavelength was kept below 0.10. All measurements were performed in triplicate (*n* = 3).

The PLQY of the CQDs was calculated using eqn (1):1
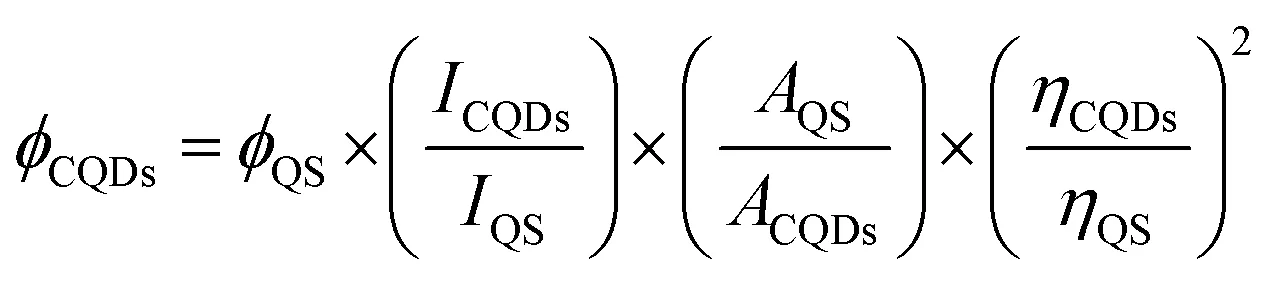
where *Φ* is the photoluminescence quantum yield, *I* is the integrated fluorescence emission intensity, *A* is the absorbance at the excitation wavelength, and *η* is the refractive index of the solvent. The subscripts CQDs and QS refer to the CQD sample and the quinine sulfate reference solution, respectively.

### 2.7. Fabrication of polymer sensor films

Polymeric sensor films were prepared by a UV-induced photopolymerization method. The photocurable resin was formulated by mixing poly(ethylene glycol) diacrylate (PEGDA 600, 45 wt%), EBECRYL® 140 (di(trimethylolpropane) tetraacrylate, Allnex, Germany, 35 wt%), and CTFA (5-ethyl-1,3-dioxane-5-yl methyl acrylate, EDMA, Arkema, France), a monofunctional acrylate monomer (20 wt%). Irgacure® 184 was added as the photoinitiator at 3 wt% relative to the combined mass of the resin components. Predetermined amounts of the synthesized fluorescent MIL-101(Fe)@CQD nanocomposite were subsequently incorporated into the resin mixture and dispersed under magnetic stirring until a homogeneous suspension was obtained. The resulting formulations were then subjected to UV irradiation to initiate photopolymerization and obtain the polymeric sensor films.

Three formulations, designated F0, F1, and F2, were prepared using different active components rather than varying active-material loadings. F0 contained no active nanomaterial, F1 contained MIL-101(Fe), and F2 contained MIL-101(Fe)@CQDs. Their compositions and roles are summarized in Table 1.

**Table 1:** Composition of the polymer composite films

Formulation	PEGDA 600 (wt%)	Ebecryl 140 (wt%)	CTFA (wt%)	MIL-101(Fe) (g)	MIL-101(Fe)@CQDs (g)
F0	45	35	20	—	—
F1	45	35	20	0.0100	—
F2	45	35	20	—	0.0100

For F1 and F2, 0.0100 g of the corresponding active material was incorporated into the photocurable formulation. Each mixture was sonicated for 1 min to improve dispersion and remove entrapped air, then transferred to a polytetrafluoroethylene mold (PTFE; Teflon®) measuring 40 mm × 12 mm × 1 mm and cured under a 300 W, 365 nm UV lamp for approximately 3 min at a lamp-to-mold distance of 10 cm. After curing, the films were immersed in deionized water for 24 h to remove residual unreacted species. The films were subsequently dried in a vacuum oven for 24 h and stored at 4 °C in the dark until further use.

### 2.8. As(iii) determination and fluorescence measurement protocol

The response of the polymer films to As(iii) was measured by fluorescence spectroscopy. A film was mounted diagonally in a quartz cuvette, with its surface oriented at approximately 45° to the incident excitation beam so that fluorescence was collected by the detector at 90° to the excitation path. The same fixed orientation was used for all measurements. Fluorescence measurements were performed at an excitation wavelength of 311 nm and monitored at an emission maximum of 450 nm. Both excitation and emission slit widths were set to 5 nm, and the photomultiplier-tube voltage was 600 V.

The effect of solution pH was evaluated before calibration, and pH 4.0 was selected for all analytical measurements. Calibration was performed over the As(iii) concentration range of 4.44 × 10^−9^–6.67 × 10^−8^ mol L^−1^. Quantitative determination of As(iii) was based on the fluorescence-intensity difference (Δ*I* = *I*_0_ − *I*), where *I*_0_ is the fluorescence intensity of the sensing film in the absence of As(iii), and *I* is the intensity measured after exposure to the analyte. The Stern–Volmer ratio (*I*_0_/*I*) was evaluated separately to characterize the concentration-dependent quenching behavior but was not used for sample quantification or for calculation of the detection and quantification limits. The limit of detection (LOD) and limit of quantification (LOQ) were calculated using 3*σ*/*m* and 10*σ*/*m*, respectively, where *σ* is the standard deviation of 10 independent blank measurements, and *m* is the slope of the Δ*I* calibration curve. Spiked samples and certified reference materials exceeding the upper calibration limit were diluted with ultrapure water before fluorescence measurement, and pre-dilution concentrations were back-calculated using the corresponding dilution factors.

For visual assessment, films immersed in As(iii) solutions were irradiated at 365 nm in a dark chamber and photographed with a digital camera. All blank, calibration, and interference measurements were performed in a sodium acetate/acetic acid buffer at pH 4.0; Na^+^ and acetate were therefore common background components rather than independent interferents. Interference experiments were conducted at 4.42 × 10^−8^ mol per L As(iii), and tolerance limits were defined by a maximum 5.0% change in Δ*I* = *I*_0_ − *I*. The same sensing film was used throughout the sequential measurement series. Between measurements, the film was regenerated by rinsing with ultrapure water for 2 min before reuse. Reusability was evaluated over 150 measurement/regeneration cycles.
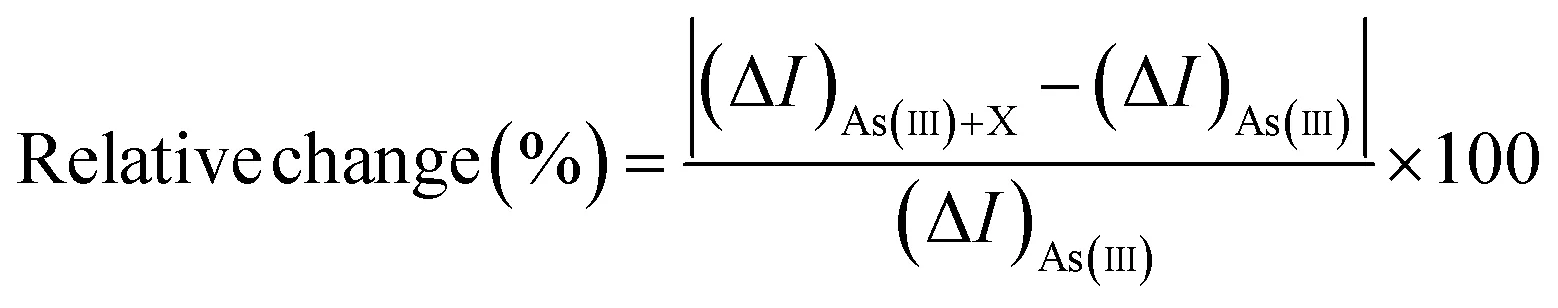
where (Δ*I*)_As(iii)_ represents the fluorescence-quenching response obtained for As(iii) alone, and (Δ*I*)_As(iii)+X_ represents the corresponding response in the presence of the potentially interfering species X. The fluorescence-quenching response was defined as Δ*I* = *I*_0_ − *I*.

### 2.9. Environmental water sample preparation and comparative HG-AAS analysis

The tap-water sample was obtained from the municipal distribution system; the lake-water sample was collected from Lake Abant in June 2026; and the groundwater sample was collected in the Yalova region of Türkiye. Before analysis, all environmental water samples were filtered through cellulose membrane filters with a 0.22 µm pore size and 47 mm diameter (Supelco 58188, Sigma-Aldrich) to remove suspended particulate matter. Filtration was performed solely for particle removal and was not used as an As(iii)/As(v) separation step. For spike-recovery experiments, known amounts of As(iii) standard were added to the environmental-water samples. The unspiked and spiked samples were analyzed using the same sample preparation and fluorescence measurement procedure. The measurement pH was adjusted to 4.0 using sodium acetate/acetic acid buffer. No digestion or preconcentration step was employed. Samples exceeding the upper limit of the calibration range were diluted with ultrapure water before fluorescence measurement, and the original concentrations were calculated using the corresponding dilution factors. Separate aliquots of the filtrate were analyzed by the proposed fluorescence method and by direct HG-AAS using an Analytik Jena ZEEnit 700 P instrument equipped with a hydride-generation system. The HG-AAS procedure was adapted from ISO 17378-2:2014. For the present As(iii)-preferential determination, the separate As(v)-to-As(iii) pre-reduction step was omitted prior to hydride generation.^36^ Arsenic absorbance was monitored at 193.7 nm with a slit width of 0.8 nm. Hydride generation employed 1.0% (w/v) NaBH_4_ stabilized in 0.1% (w/v) NaOH, with 3% (v/v) HCl as the acidic carrier and reaction medium. The HG-AAS LOD was 0.05 µg L^−1^ (0.05 ppb; 6.67 × 10^−10^ mol L^−1^), which was below the concentrations measured in the environmental-water samples. Literature reports indicate that, in the absence of a dedicated pre-reduction step, hydride formation from As(v) is suppressed relative to As(iii) under the applied conditions.^37,38^ However, omission of the pre-reduction step does not guarantee that As(v) contributes no hydride signal. Therefore, the HG-AAS procedure was calibrated with As(iii) standards and used as an operationally As(iii)-preferential comparative method under the conditions employed, rather than as evidence of exclusive As(iii) detection. Agreement with this comparator was interpreted as supporting cross-method consistency and was considered together with the independent As(v) interference study described in Section 3.3.

## 3. Results and discussion

### 3.1. Structural, morphological, and optical characterization of the sensing materials

The synthesized CQDs, MIL-101(Fe), MIL-101(Fe)@CQDs composite, and polymer sensing film were characterized by FTIR, XRD, UV-Vis, electron microscopy, DLS, and XPS.

The FTIR spectra (Fig. 1a) reveal the characteristic functional groups of each component of the developed sensing platform. The CQDs spectrum shows a broad absorption centered at approximately 3200–3500 cm^−1^, corresponding to the stretching vibrations of hydroxyl (–OH) and amino (–NH_2_) groups, confirming abundant hydrophilic surface functionalities. The absorption around 2920 cm^−1^ is attributed to the C–H stretch, while the prominent band near 1700 cm^−1^ arises from the carbonyl (C=O) stretch. These oxygen- and nitrogen-containing surface functionalities are characteristic of hydrothermally synthesized carbon quantum dots and provide active anchoring sites for interaction with the MOF framework.

**Fig. 1:**
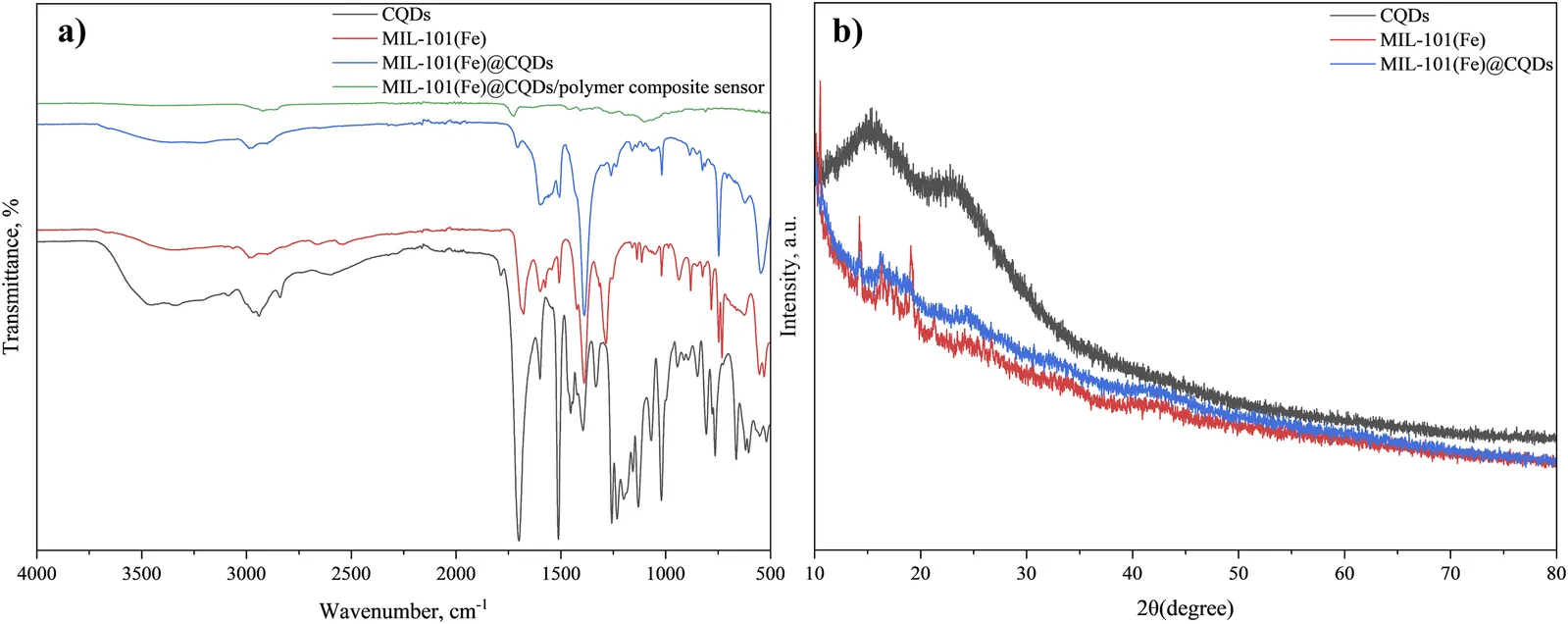
Structural characterization of the synthesized materials: (a) FTIR spectra and (b) XRD patterns of CQDs, MIL-101(Fe), and MIL-101(Fe)@CQDs.

The pristine MIL-101(Fe) spectrum exhibits characteristic vibrational bands associated with the terephthalate linker and the Fe–O coordination framework. The strong bands in the 1600–1400 cm^−1^ region are assigned to the asymmetric and symmetric stretching vibrations of coordinated carboxylate groups. In the lower-wavenumber region of the recorded spectrum (500–800 cm^−1^), the observed features are consistent with Fe–O-related vibrations in the iron-based framework. Following CQD loading, the principal framework-related vibrational bands remain detectable, suggesting that the characteristic bonding environment of MIL-101(Fe) was not substantially altered. The slight broadening in the 3200–3500 cm^−1^ region and the increased contribution of oxygen-containing groups further support the association of CQDs with MIL-101(Fe), although FTIR alone does not establish their exact spatial location. The preservation of the crystalline structure was evaluated separately by XRD.

The FTIR spectrum of the polymer sensing film exhibits a smoother profile than those of the powdered materials because the cross-linked polymer network dominates the infrared response. Nevertheless, framework- and CQD-related absorptions remain discernible, supporting the incorporation of the composite into the polymer matrix without a major detectable change in its principal chemical functionalities.

The XRD patterns in Fig. 1b show that the characteristic diffraction profile of MIL-101(Fe) was retained after CQD loading. The broad feature associated with the predominantly amorphous CQDs was also observed. No additional crystalline phase was detected within the resolution of the measurement, indicating that composite preparation did not produce a readily detectable secondary crystalline phase. The UV-Vis absorption spectrum of the CQDs (Fig. 2a) shows strong absorption below 300 nm, attributable to π → π* transitions of sp^2^ carbon domains and n → π* transitions associated with oxygen-containing surface groups. Absorption decreases markedly toward the visible region. The UV-Vis spectra of MIL-101(Fe) and MIL-101(Fe)@CQDs are shown in Fig. 2b. Both materials absorb broadly in the UV region, and the modest change in the composite spectrum is consistent with altered optical behavior after CQD loading. This spectral difference supports composite formation but does not, by itself, establish a specific charge-transfer mechanism.

**Fig. 2:**
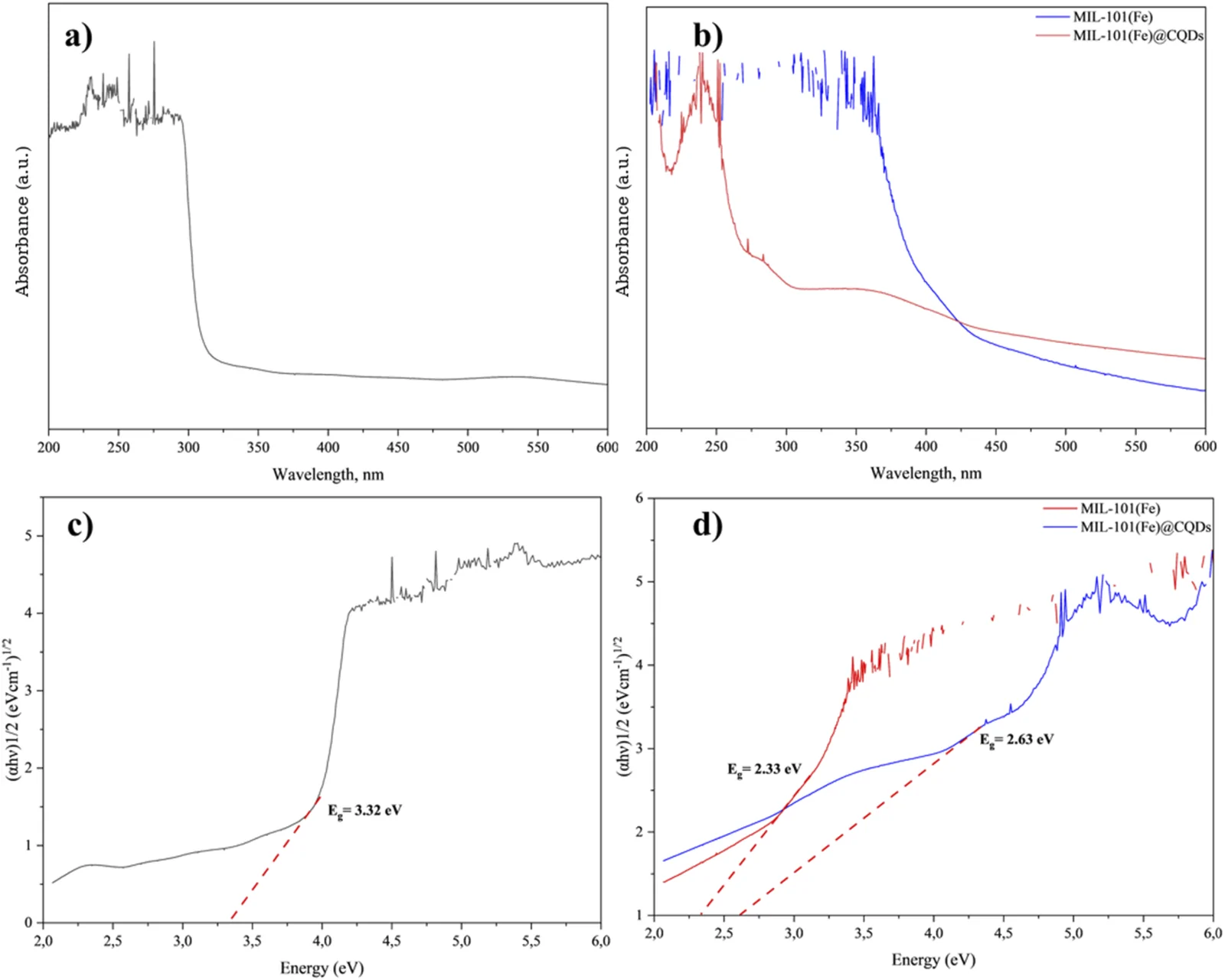
Optical characterization of the synthesized materials: (a) UV-Vis absorption spectrum of CQDs, (b) UV-Vis absorption spectra of MIL-101(Fe) and MIL-101(Fe)@CQDs, (c) Tauc plot of CQDs, and (d) Tauc plots of MIL-101(Fe) and MIL-101(Fe)@CQDs.

Apparent optical band-gap values were estimated from the *x*-axis intercepts of the linear regions of the Tauc plots constructed using the indirect allowed-transition form, (*αhν*)^1/2^*versus hν*. For the CQDs (Fig. 2c), extrapolation of the linear region yielded an apparent optical band-gap value of approximately 3.32 eV. The corresponding apparent band-gap values for MIL-101(Fe) and MIL-101(Fe)@CQDs were 2.33 and 2.63 eV, respectively (Fig. 2d). These values are interpreted as apparent Tauc band gaps for comparing the optical-transition behavior of the investigated materials. Although the difference observed after CQD loading indicates a change in the apparent optical-transition behavior, Tauc analysis alone does not establish a specific charge-transfer mechanism or a direct causal relationship with non-radiative recombination.

The morphology of the synthesized materials was investigated by scanning electron microscopy (SEM), and the results are presented in Fig. 3. As shown in Fig. 3a, pristine MIL-101(Fe) consists of well-defined polyhedral crystals with uniform particle size and sharp crystal facets, indicating successful formation of the MIL-101(Fe) framework. The highly regular morphology is consistent with the typical crystal structure reported for solvothermally synthesized MIL-101(Fe).

**Fig. 3:**
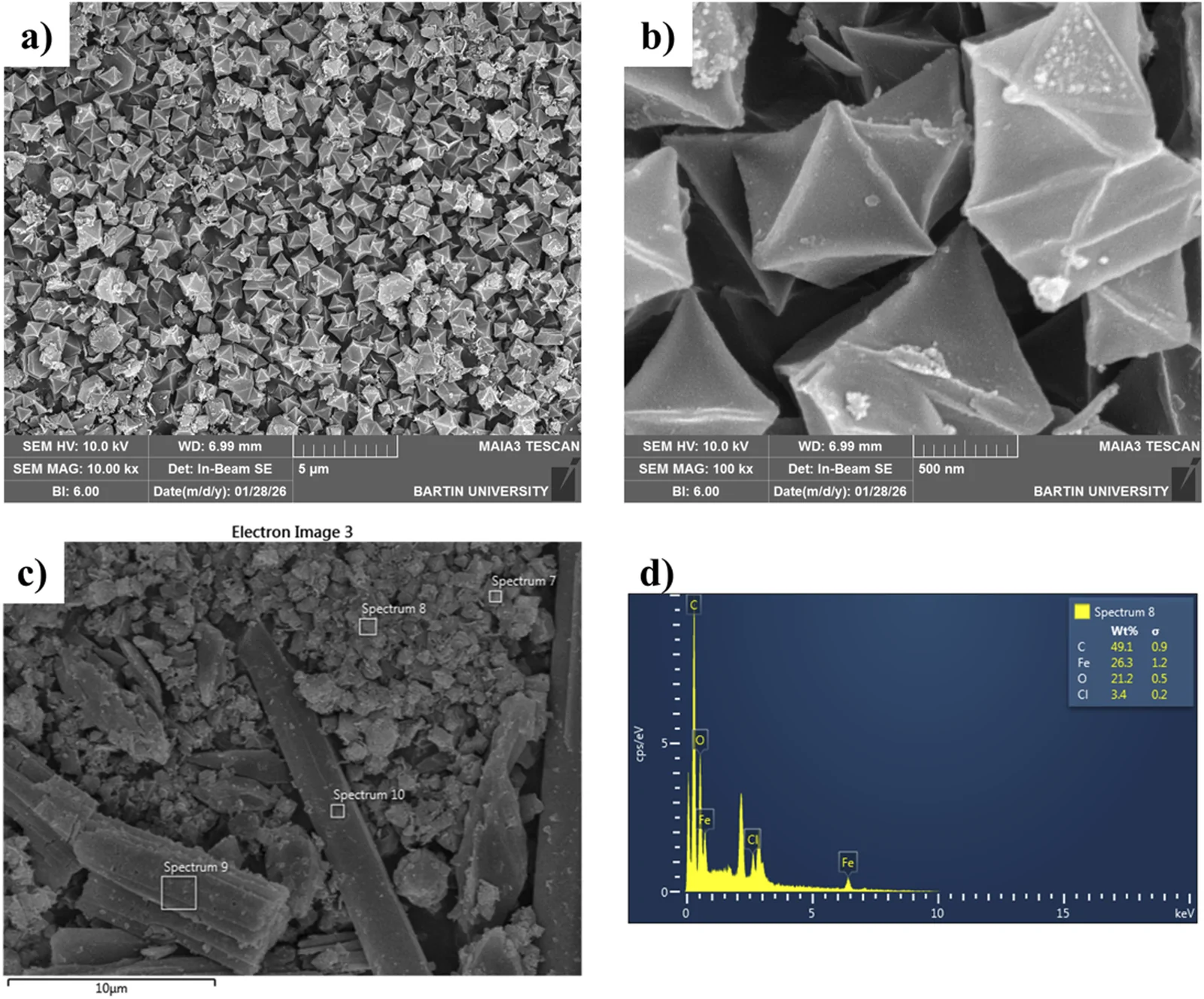
Morphological and elemental characterization of the synthesized materials: (a) low-magnification SEM image of MIL-101(Fe), (b) high-magnification SEM image of MIL-101(Fe)@CQDs, (c) SEM image of MIL-101(Fe)@CQDs with selected EDS analysis regions, and (d) corresponding EDS spectrum of MIL-101(Fe)@CQDs.

After CQD loading, the overall polyhedral morphology of MIL-101(Fe) was retained (Fig. 3b). Bright surface domains may correspond to CQD-rich regions, although their identity cannot be established from SEM contrast alone. The image is therefore consistent with surface-associated CQDs and indicates that the host-particle morphology was preserved.

The morphology of the polymer composite film is shown in Fig. 3c. The composite particles are uniformly distributed throughout the polymer matrix without obvious macroscopic aggregation, indicating effective dispersion during UV curing. This homogeneous distribution is expected to facilitate efficient interaction between the sensing sites and As(iii) during fluorescence measurements.

The elemental composition of the composite was further examined by energy-dispersive X-ray spectroscopy (EDS), as shown in Fig. 3d. The spectrum contains signals for C, O, Fe, and Cl. The C, O, and Fe signals are consistent with the principal constituents of the composite, whereas the Cl signal may originate from residual chloride associated with the iron precursor or sample preparation. The Cl signal was therefore not used as evidence of CQD loading.

The textural properties of MIL-101(Fe) and MIL-101(Fe)@CQDs were evaluated by nitrogen adsorption analysis, and the corresponding BET plots are shown in Fig. 4 and (Table 2).

**Fig. 4:**
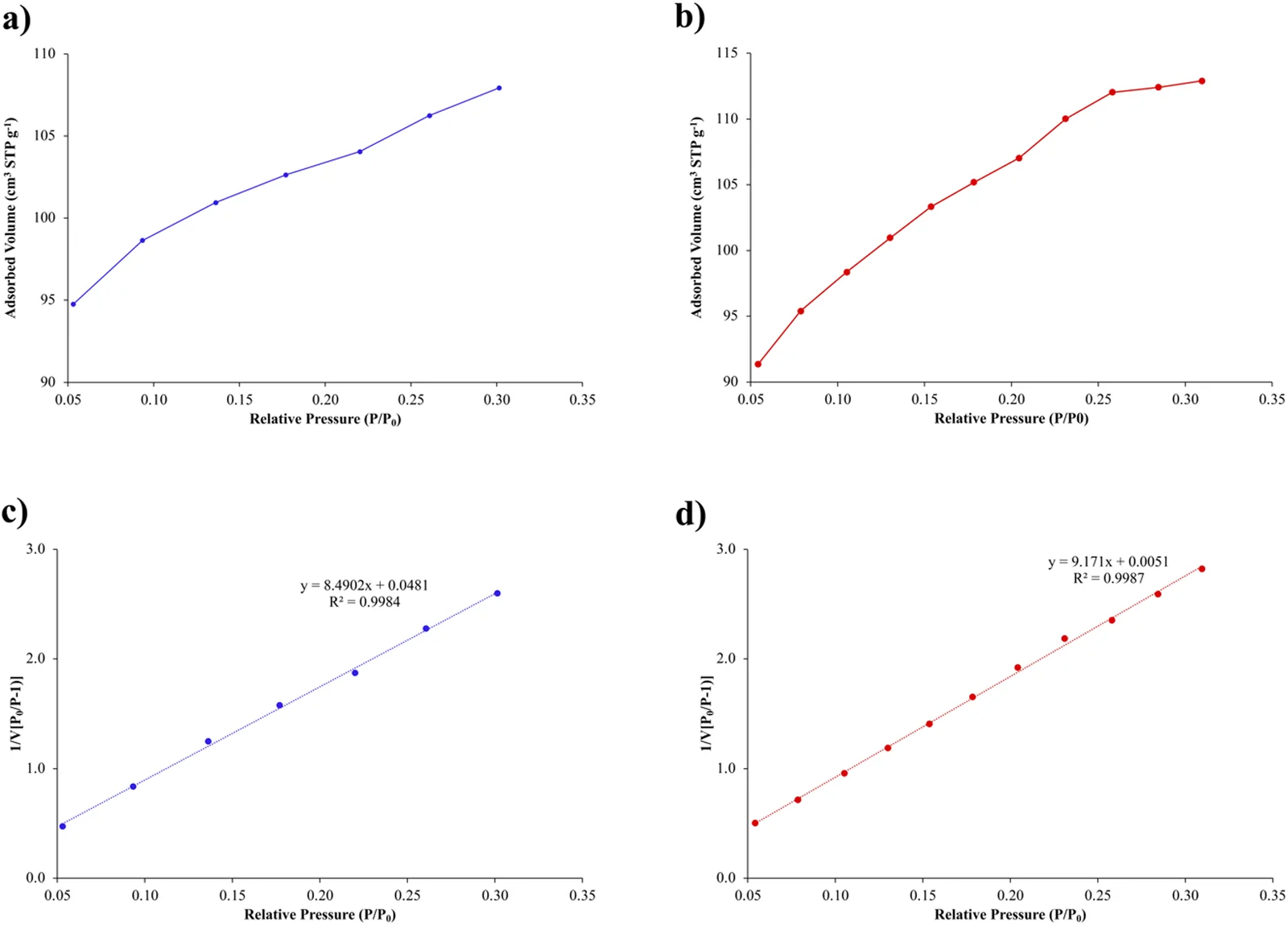
Nitrogen adsorption analysis in the BET relative-pressure region: (a) adsorption data for MIL-101(Fe), (b) adsorption data for MIL-101(Fe)@CQDs, (c) BET linear plot of MIL-101(Fe), and (d) BET linear plot of MIL-101(Fe)@CQDs.

**Table 2:** BET surface areas and linear-fit quality for MIL-101(Fe) and MIL-101(Fe)@CQDs

Sample	BET surface area (m^2^ g^−1^)	BET linear-fit *R*^2^
MIL-101(Fe)	407.87	0.9984
MIL-101(Fe)@CQDs	379.52	0.9987

As shown in Fig. 4c and d, the BET plots were highly linear for both materials. MIL-101(Fe) gave *y* = 8.4902*x* + 0.0481 with *R*^2^ = 0.9984, whereas MIL-101(Fe)@CQDs gave *y* = 9.171*x* + 0.0051 with *R*^2^ = 0.9987. The corresponding BET surface areas were 407.87 and 379.52 m^2^ g^−1^, respectively.

The BET surface area decreased slightly from 407.87 m^2^ g^−1^ for MIL-101(Fe) to 379.52 m^2^ g^−1^ for MIL-101(Fe)@CQDs. This modest reduction is consistent with partial occupation or obstruction of accessible pore space following CQD loading. Despite this slight decrease, the composite retains a highly porous architecture, indicating that composite formation did not cause a substantial loss of accessible surface area.

The morphology of the synthesized carbon quantum dots was first examined by STEM, and the corresponding image is presented in Fig. 5a. The carbon quantum dots are uniformly dispersed without noticeable aggregation, indicating that the adopted hydrothermal synthesis method produced nanoscale particles with good dispersion characteristics. Such homogeneous dispersion is beneficial for their subsequent immobilization onto the MIL-101(Fe) framework and for obtaining a uniform fluorescent sensing platform.

**Fig. 5:**
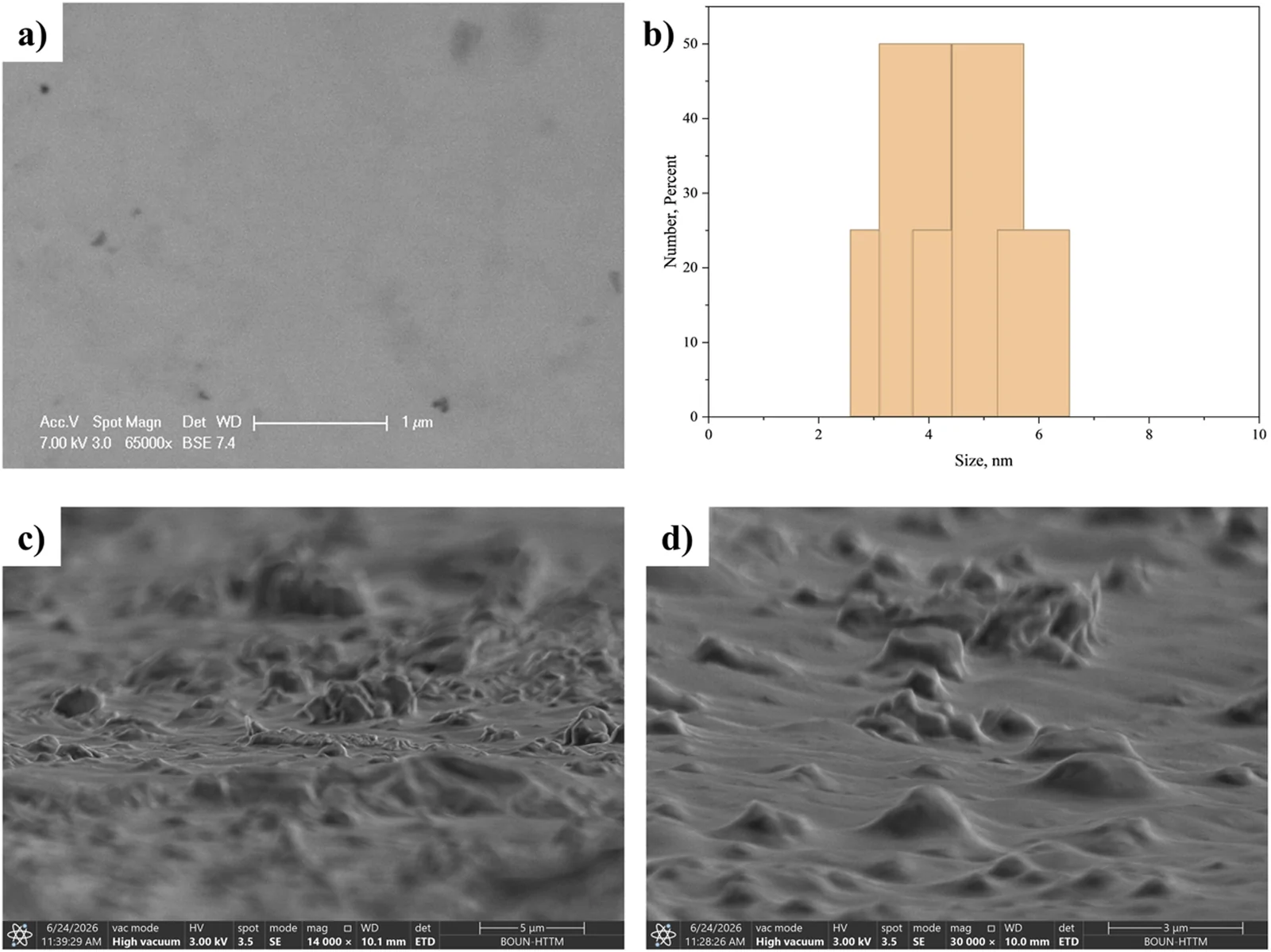
Morphological and particle-size characterization of the synthesized CQDs and polymeric films: (a) STEM image of the synthesized CQDs, (b) DLS particle-size distribution of the CQDs, (c) surface morphology of the MIL-101(Fe)@CQD/polymer composite film (F2), and (d) cross-sectional SEM image of the MIL-101(Fe)@CQD/polymer composite film (F2).

DLS analysis (Fig. 5b) revealed an average hydrodynamic diameter of approximately 3–4 nm for the synthesized CQDs. The relatively narrow particle-size distribution indicates a predominantly uniform CQD population with good dispersion under the measurement conditions.

Incorporation of the MIL-101(Fe)@CQDs into the polymer matrix resulted in a noticeable change in the film morphology, as evidenced by the surface and cross-sectional SEM images (Fig. 5c and d). The composite film exhibited a rougher and more heterogeneous morphology compared with the corresponding polymer matrix, with the MIL-101(Fe)@CQD-based composite phase distributed throughout the polymer matrix. No pronounced macroscopic phase separation or large-scale agglomeration was observed in the examined regions, indicating successful incorporation of the fluorescent composite into the polymer network. The observed morphology confirms the formation of an integrated MIL-101(Fe)@CQD/polymer composite structure suitable for subsequent fluorescence-sensing studies. The chemical composition and surface electronic states of the synthesized materials were investigated by X-ray photoelectron spectroscopy (XPS), and the results are presented in Fig. 6.

**Fig. 6:**
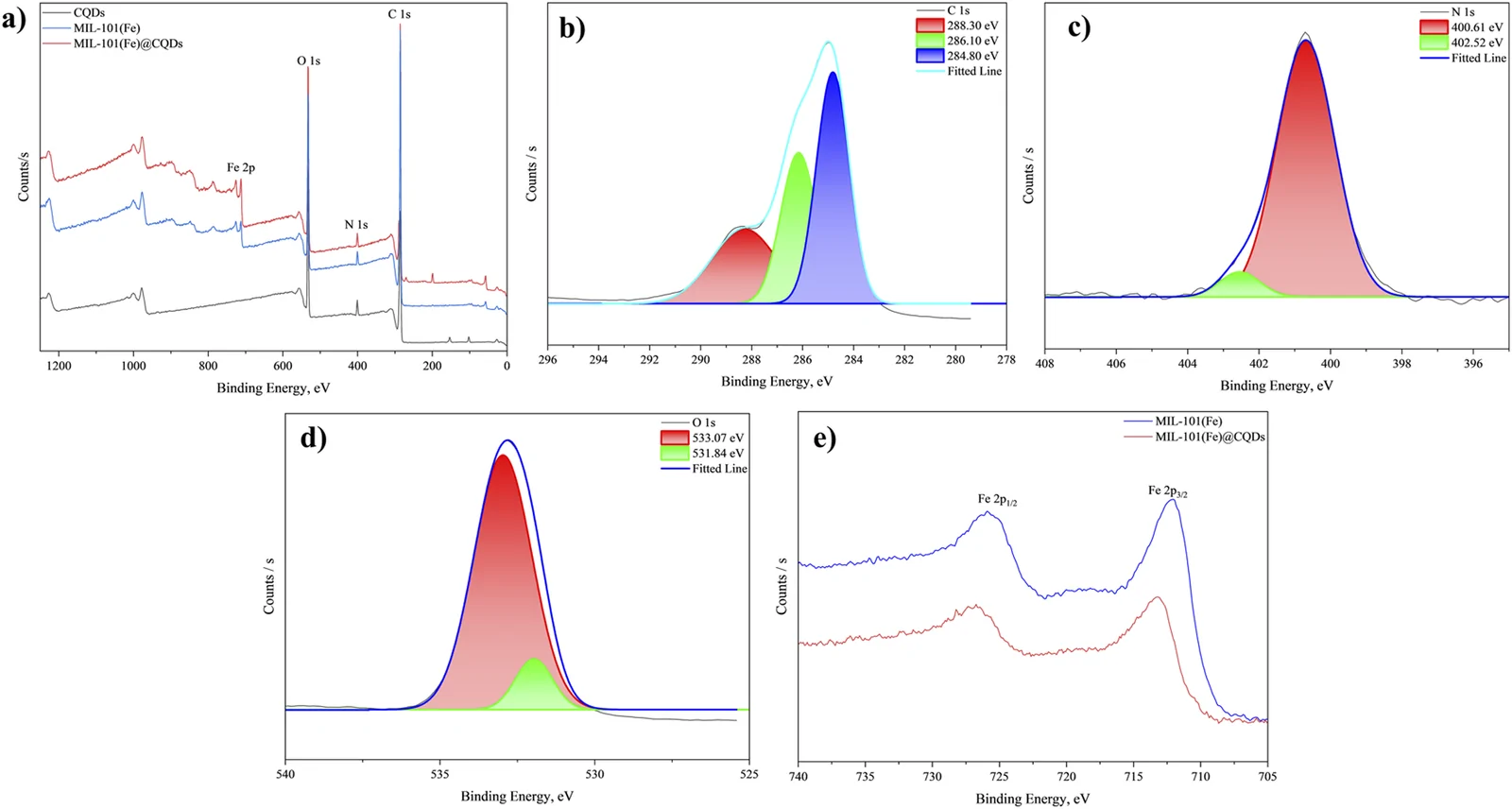
XPS characterization of the synthesized materials: (a) survey spectra of CQDs, MIL-101(Fe), and MIL-101(Fe)@CQDs; high-resolution spectra of (b) C 1s, (c) N 1s, (d) O 1s, and (e) Fe 2p.

The survey spectra (Fig. 6a) show the expected C 1s, O 1s, N 1s, and Fe 2p signals at approximately 285, 531, 400, and 711 eV, respectively. The simultaneous detection of C, O, N, and Fe supports the presence of nitrogen-containing CQDs associated with MIL-101(Fe), although the survey spectrum does not define their spatial location within or on the MOF particles. The high-resolution C 1s spectrum (Fig. 6b) was deconvoluted into three components centered at 284.80, 286.10, and 288.30 eV, assigned to C–C/C=C, C–O/C–N, and C=O/O–C=O species, respectively. The C–C/C=C component at 284.80 eV was used for charge correction. These oxygen- and nitrogen-containing functionalities are consistent with the surface chemistry of the CQDs and the terephthalate-based MIL-101(Fe) framework.

The N 1s spectrum (Fig. 6c) exhibits two contributions centered at 400.61 and 402.52 eV, assigned to amine/pyrrolic-type nitrogen and protonated or graphitic nitrogen environments, respectively. The presence of these nitrogen species is consistent with nitrogen incorporation into the CQDs synthesized using ethylenediamine.

As shown in Fig. 6d, the O 1s spectrum was deconvoluted into two components centered at 531.84 and 533.07 eV, attributed to Fe–O/carbonyl-related oxygen and C–O-containing surface functionalities, respectively. These results indicate the coexistence of the Fe-containing coordination environment and oxygenated functional groups within the composite.

The high-resolution Fe 2p spectrum (Fig. 6e) shows the characteristic Fe 2p_3/2_ and Fe 2p_1/2_ features associated with predominantly Fe(iii) centers. The absence of a major detectable shift indicates that CQD loading did not substantially alter the Fe chemical environment within the resolution of the XPS measurement.

### 3.2. Optimization of conditions for As(iii) determination

The photoluminescence (PL) properties of the developed sensing materials were systematically investigated prior to quantitative analysis. The excitation and emission spectra are shown in Fig. 7. As shown in Fig. 7a, pristine CQDs exhibit characteristic excitation and emission bands, with an emission maximum near 450 nm under UV excitation. The emission is attributed to radiative processes involving the conjugated carbon domains and surface states of the CQDs.

**Fig. 7:**
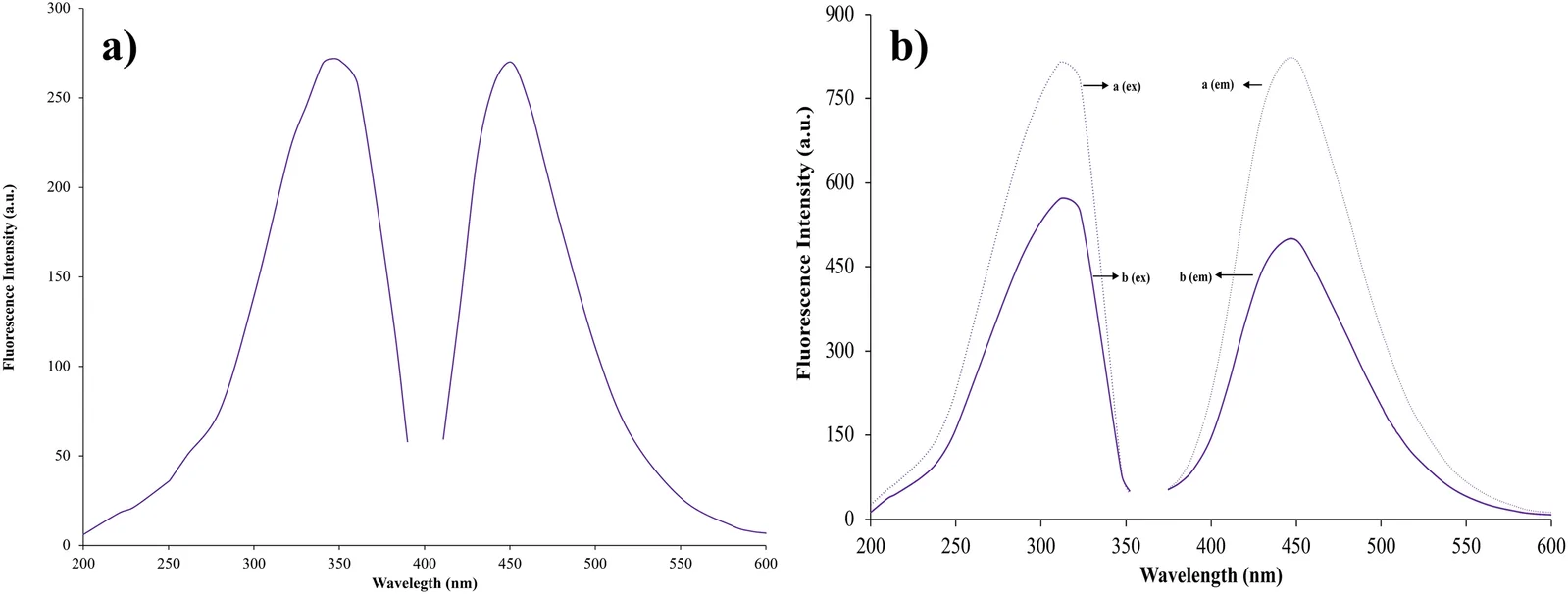
Photoluminescence properties of the sensing materials: (a) excitation and emission spectra of pristine CQDs and (b) excitation and emission spectra of the MIL-101(Fe)@CQD/polymer composite sensor (F2) before As(iii) exposure (curves a) and after As(iii) exposure (curves b).

Using quinine sulfate as the reference, the PLQY of the synthesized CQDs was 19.2 ± 3.2% (*n* = 3). This value indicates appreciable luminescence efficiency and supports the use of the CQDs as optical transducers in the fluorescence sensing film.

After loading onto MIL-101(Fe) and incorporating into the polymer matrix, the F2 film retained strong luminescence, which served as the blank response (Fig. 7b, curves a). Exposure to As(iii) produced a distinct decrease in PL intensity (Fig. 7b, curves b), demonstrating an analyte-dependent optical response. The emission maximum remained near 450 nm, indicating that the sensing response was dominated by an intensity change rather than a large shift in the dominant emissive states. The retained film fluorescence therefore provided a suitable optical signal for concentration-dependent monitoring of As(iii).

The fluorescence sensing performance of the fabricated MIL-101(Fe)@CQD/polymer composite sensor toward As(iii) was systematically investigated, and the results are summarized in Fig. 8. The excitation–emission matrix (EEM) in Fig. 8a shows a well-defined fluorescence region with a maximum emission near 450 nm, confirming that the immobilized CQDs retained a strong optical response after incorporation into the polymer matrix. For quantitative evaluation, PL responses were recorded under the optimized conditions at As(iii) concentrations of 4.44, 8.88, 13.3, 17.7, 22.2, 26.6, 31.0, 35.4, 39.9, 44.3, 48.7, 53.1, 57.5, 61.9, and 66.7 nM, corresponding to 4.44 × 10^−9^–6.67 × 10^−8^ mol L^−1^. The systematic decrease in emission intensity with increasing As(iii) concentration provided the basis for quantitative calibration of the sensing film.

**Fig. 8:**
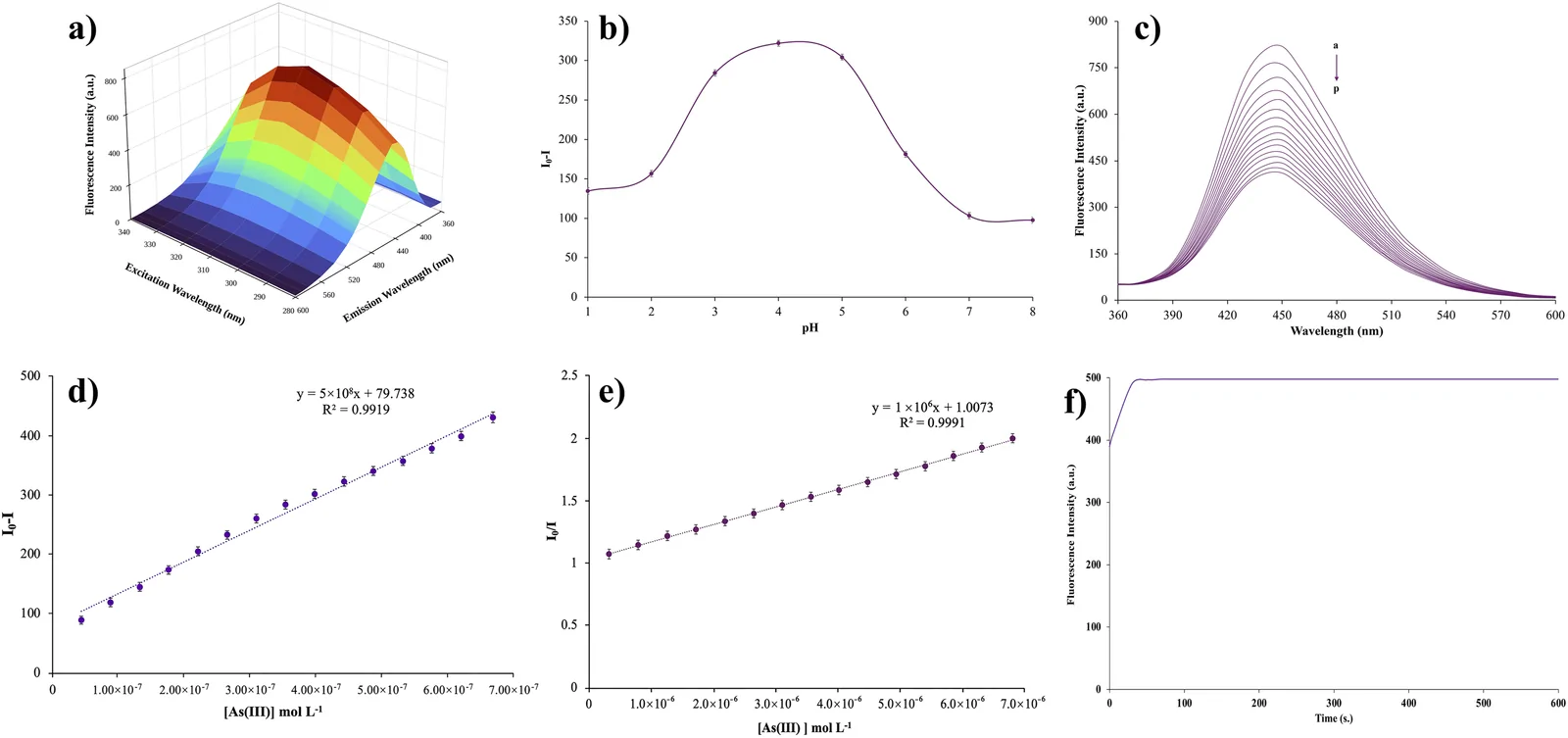
Fluorescence response of the MIL-101(Fe)@CQD/polymer composite sensor to As(iii): (a) excitation–emission matrix, (b) pH dependence in the presence of 4.43 × 10^−8^ mol per L As(iii), (c) emission spectra at increasing As(iii) concentrations, (d) calibration using *I*_0_ − *I*, (e) Stern–Volmer plot using *I*_0_/*I*, and (f) time-dependent fluorescence response.

The effect of pH on the fluorescence response of the sensing film was investigated in the presence of 4.43 × 10^−8^ mol per L As(iii), as shown in Fig. 8b. The maximum fluorescence quenching (*I*_0_ − *I*) was observed at pH 4.0, which was selected as the optimum pH for subsequent quantitative analyses. The pH-dependent behavior may reflect the combined influence of surface properties and As(iii) speciation on analyte–film interactions.

The fluorescence emission spectra recorded at increasing As(iii) concentrations are shown in Fig. 8c. The emission intensity decreased progressively with increasing As(iii) concentration, whereas the emission maximum remained nearly unchanged. This behavior indicates concentration-dependent fluorescence quenching without a substantial shift in the dominant emissive band. Considering the composite architecture, a plausible interpretation is that the Fe-containing MIL-101(Fe) framework facilitates the interaction and potential local enrichment of As(iii) near the fluorescent CQDs. Interactions between the analyte and the functional groups at the CQD surface may consequently perturb the emissive surface states and contribute to the observed fluorescence decrease. However, the available steady-state fluorescence data do not establish this interpretation as a unique mechanism. In particular, static and dynamic quenching, inner-filter effects, and electron- or energy-transfer processes cannot be distinguished unambiguously from the present measurements. Therefore, the quenching mechanism is discussed as a plausible interfacial interaction process rather than a conclusively established pathway.

The fluorescence-intensity difference (*I*_0_ − *I*) and the Stern–Volmer ratio (*I*_0_/*I*) were plotted against As(iii) concentration (Fig. 8d and e). The Δ*I* calibration response was linear over the experimentally investigated range of 4.44 × 10^−9^–6.67 × 10^−8^ mol L^−1^, with *R*^2^ = 0.9919. The Stern–Volmer plot was also linear over this range (*R*^2^ = 0.9991) and was used to characterize the quenching behavior rather than for sample quantification. Based on 10 independent blank measurements and the slope of the Δ*I* calibration curve, the LOD and LOQ were calculated as 1.49 × 10^−9^ and 4.97 × 10^−9^ mol L^−1^, respectively. The calculated LOQ is slightly higher than the lowest calibration standard (4.44 × 10^−9^ mol L^−1^); however, the calibration range describes the experimentally observed linear relationship, whereas the LOQ was independently calculated from blank variability and the calibration slope. The lowest calibration point is therefore retained as part of the calibration dataset but is not presented as an independently validated quantification limit.

The time-dependent response in Fig. 8f reached a plateau early in the monitored interval and remained stable throughout the measurement period. No significant time-dependent drift affecting the analytical response was observed under the selected acquisition conditions; therefore, measurement time did not require an additional correction or optimization step (Fig. 9).

**Fig. 9:**
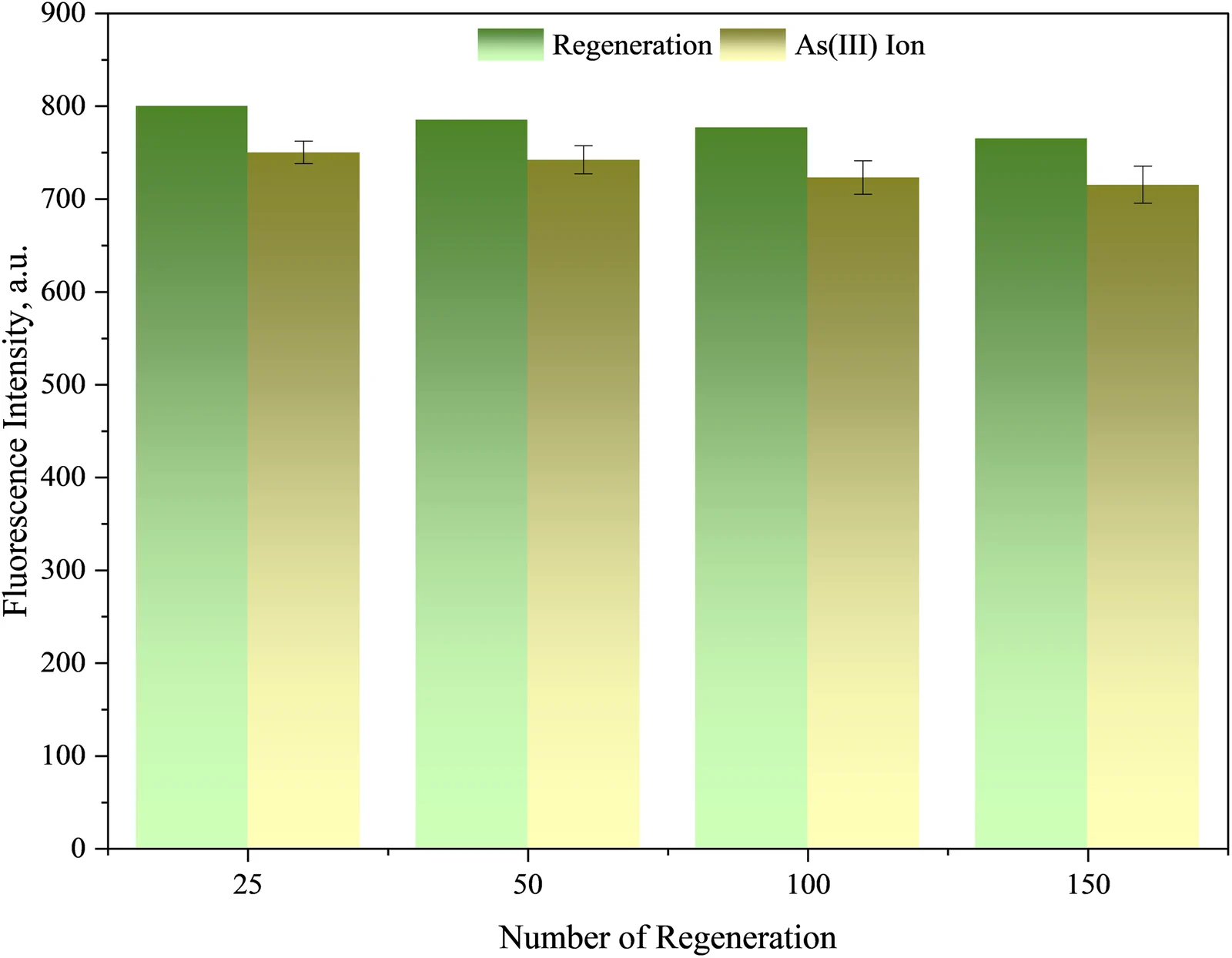
Reusability of the MIL-101(Fe)@CQD/polymer composite sensor over repeated regeneration cycles. Fluorescence intensity of the regenerated sensor before and after exposure to As(iii) at different regeneration cycles (25, 50, 100, and 150). Error bars represent the standard deviation of replicate measurements.

The same sensing film underwent 150 sequential measurement/regeneration cycles, with a 2 min ultrapure-water rinse between measurements. The response after the 150th cycle differed by only 1.4% from that of the first cycle, demonstrating strong response retention under the reuse protocol. Together with the trace-level analytical response and tolerance toward the tested species, these results support the use of the MIL-101(Fe)@CQD/polymer composite sensor as a reusable solid-state platform for As(iii) determination.

### 3.3. Selectivity and interference study for As(iii)

The selectivity of the developed MIL-101(Fe)@CQD/polymer composite sensor toward As(iii) was evaluated at a fixed As(iii) concentration of 4.42 × 10^−8^ mol L^−1^ in the presence of potentially interfering species. Interference was assessed from the direct fluorescence-quenching response, Δ*I* = *I*_0_ − *I*. The tolerance limit was defined as the highest tested concentration that produced no more than a 5.0% relative change in the response obtained for As(iii) alone. The resulting tolerance limits are summarized in Table 3.

**Table 3:** Tolerance limits for potentially interfering species in the determination of As(iii) at 4.42 × 10^−8^ mol L^−1^, based on a maximum 5.0% change in the fluorescence-quenching signal Δ*I* = *I*_0_ − *I*

Potentially interfering species	Tolerance limit (mol L^−1^)
As(v)	2.15 × 10^−5^
Sb(iii)	1.82 × 10^−5^
Sb(v)	1.05 × 10^−5^
Hg(ii)	1.13 × 10^−5^
Au(iii)	2.04 × 10^−5^
Ag(i)	1.80 × 10^−5^
Cd(ii)	9.78 × 10^−6^
Pb(ii)	1.95 × 10^−5^
Ni(ii)	3.03 × 10^−5^
Co(ii)	2.93 × 10^−5^
Cu(ii)	2.48 × 10^−5^
Cr(iii)	2.28 × 10^−5^
Fe(iii)	1.24 × 10^−5^
Mn(ii)	1.35 × 10^−5^
Al(iii)	1.49 × 10^−5^
Zn(ii)	4.93 × 10^−5^
Ba(ii)	1.20 × 10^−4^
Ca(ii)	1.01 × 10^−4^
Mg(ii)	1.29 × 10^−4^
Carbonate	2.55 × 10^−4^
Bicarbonate	2.10 × 10^−4^
Phosphate	1.15 × 10^−4^

All investigated species were tolerated at concentrations at least 220-fold higher than the As(iii) concentration. Notably, critical interfering species such as As(v), Sb(iii), and Sb(v) were tolerated at molar excesses of over 480-, 410-, and 230-fold, respectively. The lowest tolerance limit was observed for Cd(ii), which was nevertheless approximately 221 times higher than the As(iii) concentration. Ba(ii), Ca(ii), and Mg(ii) were tolerated at approximately 2715-, 2285-, and 2919-fold molar excess, respectively.

Carbonate, bicarbonate, and phosphate were tolerated at approximately 5769-, 4751-, and 2602-fold molar excess, respectively. These findings demonstrate strong resistance to interference from the tested species under the optimized experimental conditions. Because the present tolerance data are based on the analytical response, they do not, by themselves, establish the individual interaction mechanisms of the potentially interfering species with the sensing film. With particular regard to arsenic speciation, the tolerance limit determined for As(v) was 2.15 × 10^−5^ mol L^−1^ in the presence of 4.42 × 10^−8^ mol per L As(iii), corresponding to an approximately 486-fold molar excess of As(v) while maintaining a response change within the predefined 5.0% tolerance criterion. This result supports the resistance of the proposed method to As(v) interference under the tested conditions. However, because the interference experiment evaluated As(v) in the presence of As(iii), it does not independently establish the absence of a fluorescence response to As(v) alone. Therefore, the analytical response is interpreted as As(iii)-preferential under the investigated conditions rather than as proof of absolute species-exclusive detection.

All measurements were performed in a sodium acetate/acetic acid buffer. Na^+^ and acetate were therefore common background components and were not evaluated as independent interferents. Working solutions of the investigated metal species, including Sb(iii) and Sb(v), were prepared by diluting the corresponding commercially available 1000 mg L^−1^ certified AAS standard solutions with ultrapure water. The As(v) solution was prepared from analytical-grade sodium arsenate, whereas carbonate, bicarbonate, and phosphate solutions were prepared from the corresponding analytical-grade sodium salts.

### 3.4. Determination of As(iii) in environmental water samples

The applicability of the MIL-101(Fe)@CQD/polymer composite sensor was evaluated using municipal tap water filtered through a 0.22-µm cellulose membrane, lake water collected from Lake Abant in June 2026, and groundwater collected in the Yalova region. Results from the fluorescence method were compared with those obtained by the As(iii)-preferential direct HG-AAS procedure described in Section 2.9. The As(iii) spike-recovery data are summarized in Table 4.

**Table 4:** Determination of As(iii) in filtered environmental water samples by the proposed fluorescence method and direct HG-AAS, with As(iii) spike-recovery results (mean ± SD, *n* = 3)^a^

Sample	Added As(iii) (mol L^−1^)	Found by the proposed method (mol L^−1^)	Found by direct HG-AAS (mol L^−1^)	RSD of the proposed method (%)	Recovery (%)
Tap water	—	(4.50 ± 0.10) × 10^−9^	(4.40 ± 0.10) × 10^−9^	2.21	—
	1.33 × 10^−8^	(1.80 ± 0.05) × 10^−8^	(1.79 ± 0.05) × 10^−8^	2.54	101.5
	6.67 × 10^−8^	(7.29 ± 0.20) × 10^−8^	(7.25 ± 0.18) × 10^−8^	2.71	102.5
	1.34 × 10^−7^	(1.41 ± 0.05) × 10^−7^	(1.41 ± 0.04) × 10^−7^	3.26	102.1
Lake water	—	(8.50 ± 0.27) × 10^−9^	(8.60 ± 0.28) × 10^−9^	3.17	—
	1.33 × 10^−8^	(2.22 ± 0.10) × 10^−8^	(2.20 ± 0.09) × 10^−8^	4.51	103.0
	6.67 × 10^−8^	(7.70 ± 0.32) × 10^−8^	(7.68 ± 0.30) × 10^−8^	4.19	102.7
	1.34 × 10^−7^	(1.44 ± 0.06) × 10^−7^	(1.43 ± 0.06) × 10^−7^	4.02	101.3
Groundwater	—	(3.20 ± 0.12) × 10^−8^	(3.15 ± 0.11) × 10^−8^	3.71	—
	1.33 × 10^−8^	(4.56 ± 0.17) × 10^−8^	(4.55 ± 0.15) × 10^−8^	3.66	102.2
	6.67 × 10^−8^	(1.01 ± 0.04) × 10^−7^	(1.00 ± 0.04) × 10^−7^	3.65	103.4
	1.34 × 10^−7^	(1.71 ± 0.07) × 10^−7^	(1.70 ± 0.07) × 10^−7^	4.10	104.0

a The unspiked tap-water result obtained by the fluorescence method (4.50 × 10^−9^ mol L^−1^) is below the calculated LOQ of 4.97 × 10^−9^ mol L^−1^ and is therefore reported as an estimated value. The unspiked lake-water and groundwater results are above the calculated LOQ.

Environmental water samples were filtered through 0.22-µm cellulose membranes (Supelco 58188, Sigma-Aldrich) before analysis. Direct HG-AAS measurements were performed on the Analytik Jena ZEEnit 700 P under the conditions described in Section 2.9. Fluorescence results above 6.67 × 10^−8^ mol L^−1^ were obtained after dilution and back-calculation using the corresponding dilution factors.

Because the direct HG-AAS procedure did not include a separate As(v)-to-As(iii) pre-reduction step, the agreement between the two methods supports consistency of the analytical results under the applied conditions but does not independently establish exclusive As(iii) detection. The proposed fluorescence method and direct HG-AAS gave closely agreeing results within the reported experimental uncertainties. For the spiked environmental-water samples, recoveries ranged from 101.3% to 104.0%, and RSD values were below 5%. The unspiked tap-water concentration measured by the fluorescence method (4.50 × 10^−9^ mol L^−1^) was below the calculated LOQ of 4.97 × 10^−9^ mol L^−1^ and is therefore reported as an estimated value. The unspiked lake-water (8.50 × 10^−9^ mol L^−1^) and groundwater (3.20 × 10^−8^ mol L^−1^) concentrations were above the calculated LOQ.

Method accuracy was further evaluated using four certified reference materials for arsenic: NASS-5 seawater reference material from the National Research Council Canada, Seronorm Trace Elements in Serum, Level 2 from Sero/LGC Standards, and SPS-WW1 and SPS-WW2 wastewater reference materials from Spectrapure Standards AS. The results are presented in Table 5. Satisfactory agreement was observed between measured and certified arsenic values, with recoveries of 101.3–104.6% and RSD values below 5%. Together with the HG-AAS comparison and spike-recovery results for the environmental-water samples, these findings support the applicability of the sensing film for trace As(iii) determination under the tested conditions. As(iii) selectivity was assessed separately through the As(v) interference study and further supported by comparison with the As(iii)-preferential HG-AAS method.

**Table 5:** Evaluation of the proposed method using certified reference materials for arsenic^a^

Sample	Certified arsenic value ± uncertainty (mol L^−1^)	Added As(iii) (mol L^−1^)	Found value (mean ± SD, *n* = 7) (mol L^−1^)	RSD (%)	Recovery (%)
NASS-5	(1.70 ± 0.16) × 10^−8^	—	(1.70 ± 0.03) × 10^−8^	1.98	—
	(1.70 ± 0.16) × 10^−8^	1.33 × 10^−8^	(3.05 ± 0.07) × 10^−8^	2.32	101.3
	(1.70 ± 0.16) × 10^−8^	6.67 × 10^−8^	(8.54 ± 0.35) × 10^−8^	4.12	102.5
	(1.70 ± 0.16) × 10^−8^	1.34 × 10^−7^	(1.56 ± 0.06) × 10^−7^	3.85	104.2
Seronorm	(6.27 ± 0.41) × 10^−9^	—	(6.27 ± 0.15) × 10^−9^	2.45	—
	(6.27 ± 0.41) × 10^−9^	1.33 × 10^−8^	(1.99 ± 0.04) × 10^−8^	1.98	102.5
	(6.27 ± 0.41) × 10^−9^	6.67 × 10^−8^	(7.38 ± 0.17) × 10^−8^	2.32	101.3
	(6.27 ± 0.41) × 10^−9^	1.34 × 10^−7^	(1.45 ± 0.06) × 10^−7^	4.35	103.8
SPS-WW1*	(1.33 ± 0.01) × 10^−8^	—	(1.34 ± 0.05) × 10^−8^	3.91	—
	(1.33 ± 0.01) × 10^−8^	1.33 × 10^−8^	(2.72 ± 0.05) × 10^−8^	1.98	104.1
	(1.33 ± 0.01) × 10^−8^	6.67 × 10^−8^	(8.18 ± 0.19) × 10^−8^	2.32	102.5
	(1.33 ± 0.01) × 10^−8^	1.34 × 10^−7^	(1.49 ± 0.06) × 10^−7^	4.15	101.3
SPS-WW2*	(6.67 ± 0.04) × 10^−8^	—	(6.67 ± 0.15) × 10^−8^	2.32	—
	(6.67 ± 0.04) × 10^−8^	1.33 × 10^−8^	(8.02 ± 0.32) × 10^−8^	3.98	101.3
	(6.67 ± 0.04) × 10^−8^	6.67 × 10^−8^	(1.36 ± 0.03) × 10^−7^	1.98	104.6
	(6.67 ± 0.04) × 10^−8^	1.34 × 10^−7^	(2.04 ± 0.08) × 10^−7^	4.10	102.5

a RSD, relative standard deviation; SD, standard deviation. *The certified values of SPS-WW1 and SPS-WW2 correspond to the 100-fold dilution applied before analysis. CRM aliquots exceeding the upper fluorescence calibration limit of 6.67 × 10^−8^ mol L^−1^ were further diluted as required, and the reported concentrations were back-calculated using the corresponding dilution factors.

To contextualize the analytical performance of the proposed film, it was compared with selected reported As(iii) sensing systems (Table 6). The experimental linear calibration range of the proposed method was 4.44 × 10^−9^–6.67 × 10^−8^ mol L^−1^, with an LOD of 1.49 × 10^−9^ mol L^−1^ and a calculated LOQ of 4.97 × 10^−9^ mol L^−1^. The calibration range and the calculated quantification limit are reported as distinct analytical parameters. Comparisons among the listed systems should be interpreted considering differences in sensing format, detection principle, sample matrix, and validation protocol.

**Table 6:** Selected As(iii) sensing systems

Sensing material	Detection method	Linear range (mol L^−1^)	LOD (mol L^−1^)	Reference
Cys-capped CdTe QDs	Fluorometry	2.0 × 10^−9^–5.0 × 10^−7^	2.0 × 10^−9^	39
gCDs	Fluorometry	1.5 × 10^−5^–2.4 × 10^−4^	9.11 × 10^−9^	40
CQDs	Fluorometry	3.7 × 10^−7^–1.5 × 10^−3^	2.44 × 10^−8^	41
CQDs	Fluorometry	1.33 × 10^−6^–2.67 × 10^−5^	2.67 × 10^−7^	42
CQDs/f-MWCNTs/GO	Differential pulse voltammetry	1.0 × 10^−10^–1.1 × 10^−8^	5.0 × 10^−10^	43
CQDs	Fluorometry	2.0 × 10^−9^–1.2 × 10^−8^	2.3 × 10^−9^	44
GSH/DTT-CdTe QDs	Fluorometry	6.67 × 10^−8^–1.33 × 10^−6^	2.27 × 10^−8^	45
BN-CQDs	Fluorometry	1.34 × 10^−10^–1.34 × 10^−7^	1.34 × 10^−9^	46
MIL-101(Fe)@CQDs/polymer film	Fluorometry	4.44 × 10^−9^–6.67 × 10^−8^	1.49 × 10^−9^	This work

The free-standing polymer film eliminates the need for nanoparticle separation and enables straightforward positioning during fluorescence measurement. The same film was reused for over 150 measurement/regeneration cycles, with a 2 min ultrapure-water rinse between measurements; the response after the final cycle differed by 1.4% from the first-cycle response. The film format is also compatible with future compact fluorescence readout, although field deployment was not evaluated in the present study.

Overall, the comparison indicates that the MIL-101(Fe)@CQDs/polymer composite sensor combines trace-level fluorescence response with an easy-to-handle solid-state format.

## 4. Conclusion

In this study, a UV-crosslinked MIL-101(Fe)@CQD/polymer composite sensor was developed as a free-standing fluorescent platform for As(iii)-preferential fluorescence determination under the tested conditions. FTIR, XRD, UV-Vis spectroscopy, BET, SEM/STEM, DLS, and XPS confirmed composite formation and its incorporation into the polymer film. The synthesized CQDs exhibited a PLQY of 19.2 ± 3.2% (*n* = 3). Under the optimized conditions, the Δ*I* calibration response was linear over the experimentally investigated concentration range of 4.44 × 10^−9^–6.67 × 10^−8^ mol L^−1^, with *R*^2^ = 0.9919. The calculated limit of detection (LOD) and limit of quantification (LOQ) were 1.49 × 10^−9^ and 4.97 × 10^−9^ mol L^−1^, respectively. The experimental calibration range and the calculated LOQ are reported as distinct analytical parameters; the lowest calibration standard is retained as part of the calibration dataset but is not presented as an independently validated quantification limit. The Stern–Volmer plot showed *R*^2^ = 0.9991 and was used only to characterize quenching behavior, rather than for sample quantification. At an As(iii) concentration of 4.42 × 10^−8^ mol L^−1^, all tested potentially interfering species were tolerated at concentrations at least approximately 221-fold higher than the As(iii) concentration. Spike-recovery tests in environmental-water samples yielded recoveries of 101.3–104.0%, with RSD values below 5%. The certified reference material (CRM) spike-recovery tests yielded recoveries of 101.3–104.6%, also with RSD values below 5%. The unspiked tap-water result (4.50 × 10^−9^ mol L^−1^) is reported as an estimate because it falls below the calculated LOQ. Environmental-water results were consistent with direct HG-AAS performed without a separate As(v)-to-As(iii) pre-reduction step. This agreement supports cross-method consistency under the applied conditions but does not independently establish exclusive As(iii) detection. Samples exceeding the upper calibration limit were analyzed after controlled dilution, and their original concentrations were calculated using the corresponding dilution factors. The same sensing film retained its response over 150 measurement/regeneration cycles, with only a 1.4% difference between the first and final responses. The integration of MIL-101(Fe), CQDs, and a UV-curable polymer matrix provides an easy-to-handle solid-state platform for trace As(iii) determination. The results support further development of polymer-supported fluorescence films for As(iii)-preferential analysis under the stated measurement conditions, including further evaluation of long-term reuse and compact instrumental readout.

## Author contributions

Y. E. T.: investigation, methodology, formal analysis, data curation, validation, visualization, writing – original draft. S. Ç.: conceptualization, methodology, validation, formal analysis, writing – original draft, writing – review and editing. M. V. K.: conceptualization, supervision, resources, project administration, writing – review and editing. All authors reviewed and approved the final manuscript.

## Conflicts of interest

The authors declare no conflict of interest.

## Data Availability

The data that support the findings of this study are available from the corresponding author upon reasonable request.
